# Evolution of Cooperation in a Heterogeneous Graph: Fixation Probabilities under Weak Selection

**DOI:** 10.1371/journal.pone.0066560

**Published:** 2013-06-20

**Authors:** Cong Li, Boyu Zhang, Ross Cressman, Yi Tao

**Affiliations:** 1 Key Lab of Animal Ecology and Conservational Biology, Institute of Zoology, Chinese Academy of Sciences, Beijing, P.R. China; 2 School of Mathematical Sciences, Beijing Normal University, Beijing, P.R. China; 3 Department of Mathematics, Wilfrid Laurier University, Waterloo, Ontario, Canada; University of Maribor, Slovenia

## Abstract

It has been shown that natural selection favors cooperation in a homogenous graph if the benefit-to-cost ratio exceeds the degree of the graph. However, most graphs related to interactions in real populations are heterogeneous, in which some individuals have many more neighbors than others. In this paper, we introduce a new state variable to measure the time evolution of cooperation in a heterogeneous graph. Based on the diffusion approximation, we find that the fixation probability of a single cooperator depends crucially on the number of its neighbors. Under weak selection, a cooperator with more neighbors has a larger probability of fixation in the population. We then investigate the average fixation probability of a randomly chosen cooperator. If a cooperator pays a cost for each of its neighbors (the so called fixed cost per game case), natural selection favors cooperation if the benefit-to-cost ratio is larger than the average degree. In contrast, if a cooperator pays a fixed cost and all its neighbors share the benefit (the fixed cost per individual case), cooperation is favored if the benefit-to-cost ratio is larger than the harmonic mean of the degree distribution. Moreover, increasing the graph heterogeneity will reduce the effect of natural selection.

## Introduction

Evolution of cooperation is one of the most important theoretical questions in evolutionary biology and social biology [1]–[4]. In the framework of evolutionary game theory, cooperation has been studied in a variety of game theoretical models such as the Prisoner's Dilemma [1]–[3]. It is well known that in unstructured populations, natural selection favors defectors over cooperators [2]–[3]. This means that in a population where all individuals have the same chance to interact with each other, defectors will have a higher average payoff than cooperators (i.e. natural selection will increase the relative abundance of defectors and drive cooperators to extinction). However, in more realistic populations, different individuals interact with different subsets of the whole population. This kind of structure can be described by means of complex networks (or graphs), in which players of an evolutionary game occupy the vertices of a network, and the edges denote the links between individuals in terms of game dynamical interaction.

A great deal of research has been devoted to explain the influence of network structure on the evolution of cooperation. Pioneering work by Nowak and May [5] unveiled that a spatial lattice where each player interacts only with his/her four (or eight) immediate neighbors is a viable mechanism for the emergence of cooperation. The success of their model has attracted considerable attention, subsequent studies including not only theoretical investigations [6]–[14] but also economic experiments [15]–[17]. In these studies, the spatial structure retains homogeneity (or regularity) since every individual is topologically identical to any other individual in the population. However, empirical evidence shows that most networks related to interactions in the real world are heterogeneous in that some individuals have many more neighbors (i.e. have higher degree) than others [18]–[23]. Representative networks include the random network [24]–[25], Small-World network [26]–[27] and scale-free network [28]–[29]. Many recent researches have revealed that graph heterogeneity dramatically enhances cooperation in complex networks [30]–[36], in particular for the scale-free network [37]–[42]. Although there are many differences in the models considered in this literature (e.g. different update rules, payoff structure, network clustering, etc.), one of the main reasons behind the increase of cooperation levels in the scale-free network is that hubs are usually occupied by cooperators, which ensures their long term success in the evolutionary process. The same occurs when the network structure is coevolving together with the strategy dynamics [43]–[48]. If neighboring pairs of cooperators are more stable than pairs that include at least one defector, cooperators are more likely to have higher degree, which gives rise to highly cooperative heterogeneous networks.

Recently, Ohtsuki et al. [49] extended the approach of Nowak and May [5] and developed a theoretical model based on the simplified Prisoner's Dilemma (PD) game to investigate the evolution of altruistic cooperation on a graph (or a social network) where a donor pays a cost, 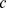, for the recipient to get a benefit, 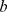. They alleged that natural selection favors the emergence of cooperation if the benefit of the altruistic act, 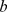, divided by the cost, 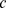, exceeds the average degree of the graph, 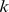, i.e. 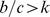. They considered a population of 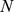 individuals consisting of cooperators and defectors (where the population size 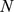 is fixed), and assumed that: (i) a cooperator helps all individuals to whom it is connected, i.e. if a cooperator is connected to 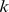 other individuals and 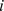 of those are cooperators, then its payoff is 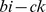; (ii) a defector does not provide any help, but it can receive the benefit from neighboring cooperators, i.e. if a defector is connected to 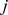 cooperators, then its payoff is 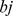; (iii) the fitness of an individual is given by its baseline fitness plus the payoff, where strong (weak) selection means that the payoff is large (small) compared to the baseline fitness; and (iv) for the evolutionary dynamics, in each time step, a random individual is chosen to die, and the neighbors compete for the empty site proportional to their fitness (this process is called ‘death-birth’ updating). Ohtsuki et al.'s theoretical results show that if all individuals have the same number of neighbors, denoted by 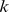, then the fixation probability of a single cooperator exactly equals 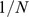 if there is no selection, and, under weak selection, it is larger than 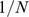 if 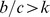.

However, Ohtsuki et al.'s theoretical analysis [49] is based only on a regular graph where all vertices have the same degree. Numerical simulations in their paper show that the fixation probabilities of cooperators in heterogeneous graphs deviate from the theoretical prediction. Thus, a more challenging question is to derive the relation between the fixation probability and the graph heterogeneity. In general, the heterogeneity of a graph (or a social network) can be described by the mean and variance of its degree distribution [34], [50]. The degree distribution of a graph, denoted by 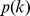, gives the frequency of vertices with degree 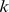 for 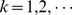, or, alternatively, 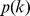 is the probability that a randomly chosen individual has exactly 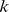 neighbors.

In this paper, we develop and analyze the connection between 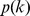 and the fixation probabilities of cooperators when selection is weak that provides a new theoretical explanation for the effect of graph heterogeneity on the evolution of cooperation.

## Methods

**Figure 1:**
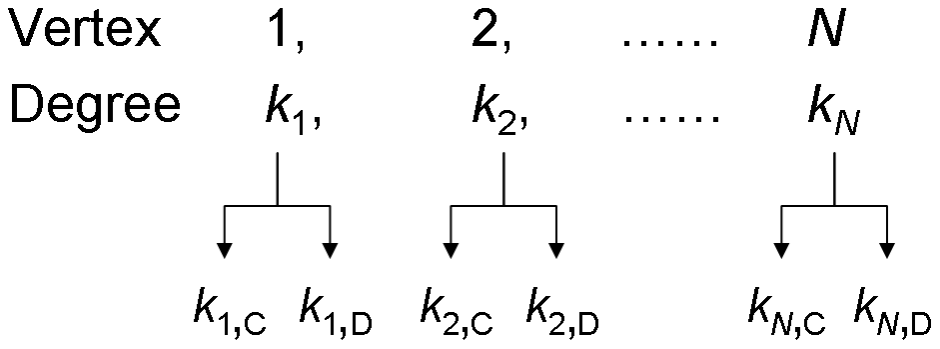
The vertices and degrees in a heterogeneous graph. The 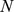 vertices are labeled vertex 1, vertex 2, 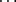, vertex 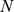. The degree of vertex 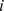 is 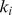, and 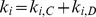 where 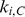 is the number of 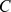-neighbors and 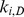 the number of 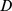-neighbors.

Consider a connected graph with 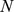 vertices and with degree distribution 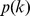. Similar to Ohtsuki et al. [49], the individual at each vertex is either a cooperator in interactions with all of the neighbors or a defector. We label these 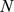 vertices as vertex 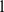, vertex 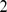, 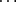, vertex 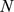, respectively, and the degree of vertex 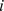 is denoted by 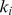 for 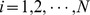. For an individual at vertex 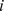, its degree 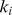 can be also expressed as 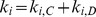 where 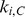 is the number of its neighbors with strategy 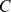 (cooperation) and, similarly, 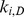 the number of its neighbors with strategy 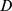 (defection) (see Figure 1). The average degree of the graph, denoted by 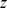, is 
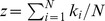
, or 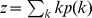. On the other hand, the strategy of the individual at vertex 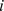 is denoted by 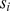 with 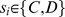 for 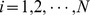, i.e. if this individual is a cooperator (or a defector), then 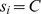 (or 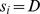).

In our model, if there exists an edge between two vertices 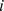 and 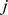 for 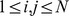, then we use 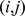 to denote a directed edge from vertex 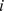 to vertex 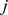. For the interaction between two individuals at vertices 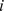 and 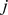, we consider the two directed edges 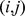 and 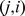 equivalent. Notice that the total number of the directed edges in the graph is 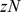. Thus, the proportion of the directed edges starting from the cooperators in the total directed edges, denoted by 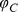, can be given by 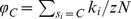, i.e. 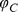 denotes a ratio of the total degree of cooperators to the total number of directed edges. Similarly, the proportion of directed edges starting from the defectors, denoted by 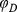, is 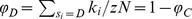. It is easy to see that if 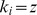 for all 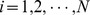 (i.e. all vertices have the same degree), then 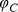 exactly represents the proportion of cooperators in the population (i.e. the frequency of strategy 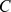 in the population). For a general heterogeneous graph, we will use the time evolution of 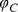 to measure the evolution of cooperation.

We also assume that an individual is randomly chosen to die, and its neighbors compete for the empty site proportional to their fitness, i.e. the ‘death-birth’ updating process [49], [51]–[52]. For a randomly chosen defector, let 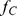 denote the fitness of its 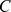-neighbors and 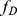 the fitness of its 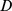-neighbors. These are given by 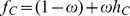 and 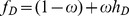, respectively, where the parameter 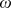 measures the intensity of selection with 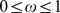 [49], [51]–[52], and 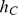 (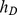) is the expected payoff of its 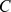-neighbors (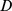-neighbors). Similarly, for a random chosen cooperator, let 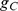 denote the fitness of its 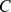-neighbors and 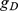 the fitness of its 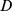-neighbors, which are 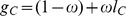 and 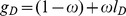, respectively, where 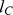 (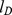) is the expected payoff of its 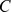-neighbors (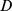-neighbors).

For the expected payoffs of both 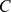
- and 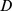-individuals, two cases are considered in our model, called the fixed cost per game and the fixed cost per individual, respectively [33]. For the fixed cost per game, a cooperator pays a cost 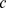 for each of its neighbors to get a benefit 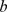 [49]; and for the fixed cost per individual, a cooperator with 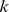 neighbors pays a cost 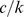 for each of its neighbors to get a benefit 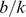, i.e. a cooperator will provide a combined fixed benefit 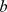 to all its neighbors and pay a fixed cost 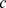 equally shared by all its neighbors [33]. Notice also that the conditional probability that a neighbor of a 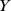-individual is a 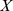-individual is given by 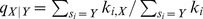 for 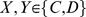. Thus, for a randomly chosen 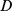-individual with degree 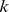, the expected payoffs of its 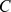
- and 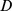-neighbors are 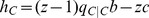 and 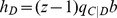, respectively, in the case of fixed cost per game. In the case of fixed cost per individual, these expected payoffs are 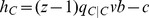 and 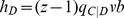, respectively, where 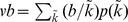 is the expected benefit from a 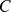-neighbor (see Supporting Information S1). Similarly, for a randomly chosen 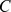-individual with degree 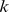, the expected payoffs of its 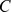
- and 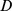-neighbors are 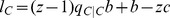 and 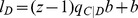, respectively, in the case of fixed cost per game, and 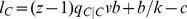 and 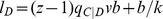, respectively, in the case of fixed cost per individual (see Supporting Information S1).

If a defector with degree 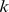 is randomly chosen to die, then the probability that this individual has exactly 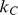 neighbors with strategy 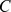 and 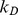 neighbors with strategy 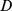 is 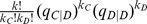. Thus, the probability that the change of 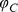 equals exactly 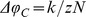 in one time step is 
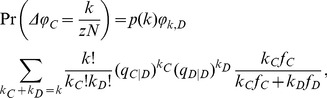
(1)


where 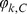 (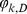) denotes the proportion of vertices with degree 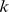 that are in strategy 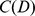. Similarly, if a cooperator with degree 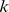 is randomly chosen to die, then the probability that the change of 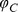 equals exactly 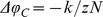 is 
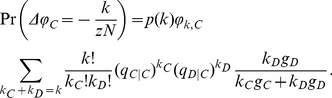
(2)


We next derive the time evolution of 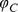 under weak selection. Weak selection means that the payoff from the game represents only a small contribution to fitness [2]. In a heterogeneous graph, this requires that the payoff for the player with highest degree is small compared to the baseline fitness, i.e., 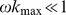, where 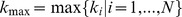. In the limit of weak selection, the expected change of 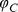, denoted by 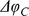, in the time interval 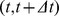 is approximated by 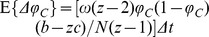 for the fixed cost per game and 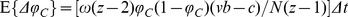 for the fixed cost per individual (this follows from Eq (1) and Eq (2) as shown in Supporting Information S1). For both cases, the variance of 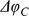 is 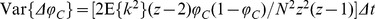 (see also Supporting Information S1). So, from the theory of diffusion approximation [53], if the initial value of 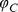 is 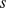 (with 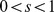), then the fixation probability of cooperation, denoted by 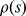, is 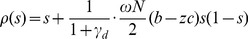
(3)for the case of fixed cost per game, and, similarly,
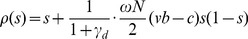
(4)for the case of fixed cost per individual (the mathematical proofs of these two equations are shown in Supporting Information S1). In both Eq.(3) and Eq.(4), 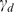 is the square of the relative deviation of the degree distribution (i.e. 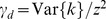 where 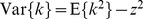 is the variance of the degree distribution) and provides a measure of heterogeneity for the graph.

## Results

**Figure 2:**
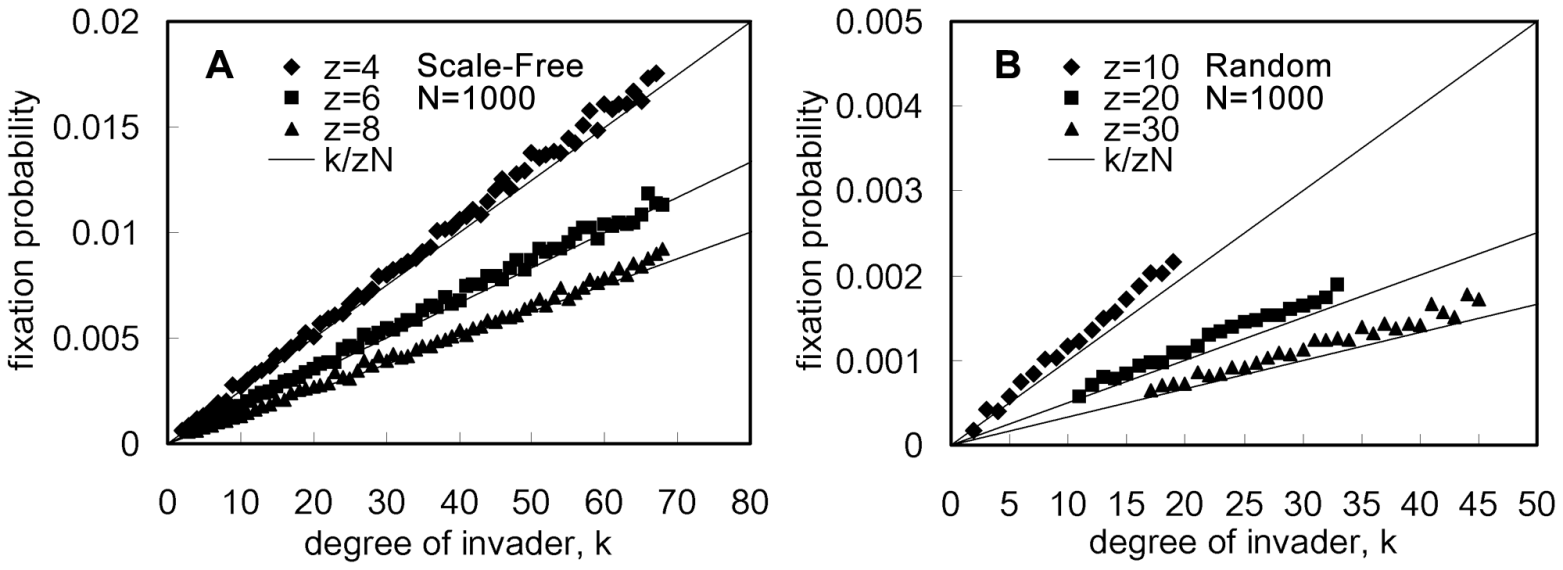
Effect of an individual's degree on the fixation probability under neutral selection. The scale-free network (generated according to [28]) and the random graph (generated according to [62]) are used to test the effect of graph heterogeneity on neutral selection (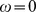). For each of these two graphs, the total population size is 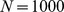 and the fixation probability of a single cooperator is measured using the fraction of runs where cooperators reached fixation out of 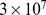 runs (based on 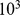 graphs and 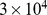 runs per graph). The simulation results are plotted in Figure 2A for the scale-free network and in Figure 2B for the random graph. For both Figures 2A and 2B, the 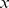-axis denotes the number of a single cooperator's neighbors and the 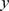-axis the fixation probability of cooperation. In each of Figures 2A and 2B, the three solid lines represent the theoretical predictions of fixation probabilities for three average degrees, where 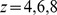 in Figure 2A and 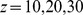 in Figure 2B, and diamonds, squares and triangles denote the simulation results. It is clear that the simulation results match the theoretical prediction well.

It is easy to see that for both Eq.(3) and Eq.(4), if 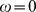, then 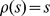. That is, under neutral selection, the fixation probability of a single cooperator with degree 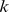 exactly equals 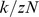. To test the effect of graph heterogeneity on neutral selection, two graphs are considered in Figure 2; namely, the scale-free network and the random graph. The simulation results match the theoretical prediction well: if there is no selection, then the fixation probability of a single cooperator is uniquely determined by the number of its neighbors. In a heterogeneous graph, if there is no selection, then the fixation probability of a single cooperator with degree 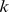 is larger (less) than 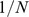 if 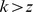 (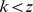). This implies that the condition that the fixation probability of a randomly chosen cooperator is larger (or less) than 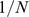 cannot be used as a criterion to estimate whether cooperation will be favored by natural selection in a general heterogeneous graph, the degree of the cooperator must also be considered. This is different from the situation for a homogeneous graph [49].

**Figure 3:**
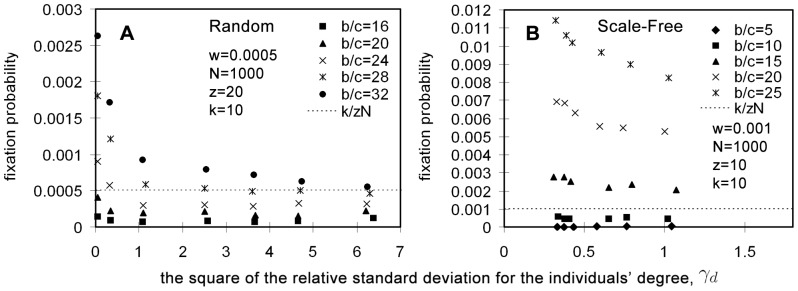
Effect of graph heterogeneity on the fixation probability under weak selection and fixed cost per game. Simulation results for the fixation probability in random graphs and scale-free graphs with different heterogeneities are shown in Figure 3A and 3B, respectively (see the generation of these graphs in Supporting Information S1). In Figure 3A, 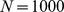 and 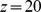. The benefit-to-cost ratio is taken as 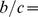 16, 20, 24, 28 and 32, respectively, and the selection intensity is 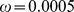. In Figure 3B, 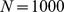 and 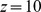. The benefit-to-cost ratio is taken as 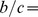 5, 10, 15, 20 and 25, respectively, and the selection intensity is 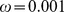. In both Figures 3A and 3B, the 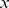-axis denotes 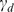 and the 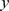-axis the fixation probability of a single cooperator with 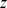 neighbors. The fixation probability is measured using the fraction of runs where cooperators reached fixation out of 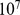 runs. The dash line 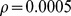 in Figure 3A and the dash line 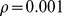 in Figure 3B denote respectively the fixation probability under neutral selection (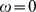). Both Figures 3A and 3B show the tendency that for all different values of 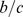, fixation probability approaches that of neutral selection as 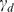 increases.

When there is weak selection (i.e. 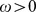 but 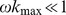), the term 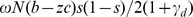 in Eq.(3) denotes the effect of natural selection on cooperation for the case of fixed cost per game. In particular, cooperation will be favored by natural selection (i.e. 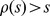) if the benefit-to-cost ratio 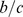 is larger than the average degree 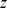 of the graph. Although this result is similar to Ohtsuki et al. [49] (obviously, if all individuals have the same degree, then 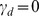 and Eq.(3) is exactly the same as Ohtsuki et al.'s theoretical result), we also notice that the term 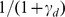 represents how selection favoring cooperation is affected by the graph heterogeneity. Specifically, for given average degree 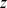 and weak selection parameter 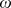, the absolute size of the term 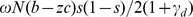 will decrease with the increase of 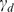. That is, an increase in graph heterogeneity will reduce the effect of selection. Simulation results in Figure 3 show this tendency. The fixation probability approaches that of neutral selection as 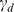 increases.

**Figure 4:**
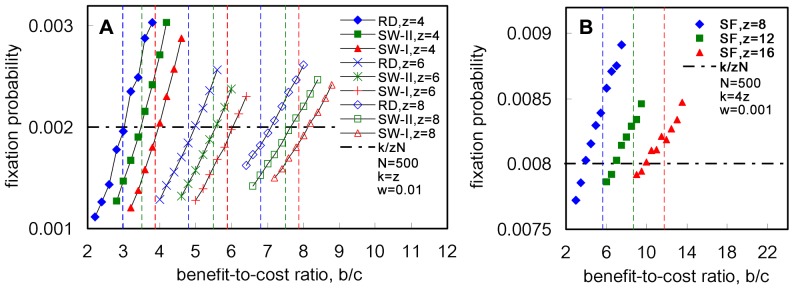
Effect of the fixed cost per individual on the fixation probability under the weak selection. Four heterogeneous graphs, SW-I (the Small-World graph generated according to [27] with rewiring probability 0.1), SW-II (the Small-World graph generated according to [27] with rewiring probability 1), RD (random graph generated according to [62]) and SF (scale-free graph generated according to [28]) are used to test the theoretical predictions. Simulation results for the fixation probability of a single cooperator with 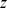 neighbors in SW-I, SW-II and RD are shown in Figure 4A. For 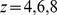, the harmonic means of the degree distribution are 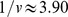, 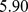, 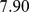 in SW-I, 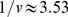, 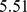, 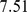 in SW-II, and 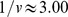, 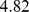, 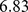 in RD, respectively. The vertical dash line represents the harmonic mean of degree distribution, 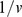, for each of 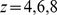, where the red, green and blue vertical dash lines correspond to SW-I, SW-II and RD, respectively. Figure 4B shows the simulation results for the fixation probability of a single cooperator with 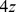 neighbors in SF. For 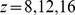, the harmonic means of the degree distribution are 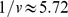, 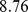, 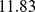, respectively. The vertical dash line represents the harmonic mean of degree distribution, 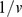, where the blue, green and red vertical dash lines correspond to 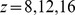, respectively. In both Figures 4A and 4B, the 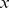-axis denotes the benefit-to-cost ratio, 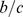, the 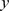-axis the fixation probability, and the horizontal dash-point line denotes the fixation probability of a single cooperator under neutral selection (i.e. 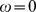) which is 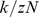. The fixation probability of a single cooperator is measured using the fraction of runs where cooperators reached fixation out of 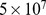 runs (based on 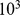 graphs and 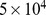 runs per graph). Both Figures 4A and 4B show that the theoretical predictions present a good approximation to the numerical results.

Similar to Eq.(3), 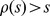 if 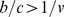 in Eq.(4) for the case of fixed cost per individual where 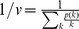 is the harmonic mean of the degree distribution. Since 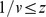 with equality if and only if the graph is homogeneous, if selection is weak, then cooperation should be more easily favored by natural selection in a heterogeneous graph. To examine whether this theoretical prediction is true, simulations for four graphs are considered: two Small-World networks (denoted by SW-I and SW-II), a random graph (denoted by RD) and a scale-free graph (denoted by SF). For all four graphs, the total population size is taken as 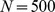 and three different average degrees are considered. The simulation results are plotted in Figure 4, which show the fixation probability of a single cooperator in the case of fixed cost per game for different values of the benefit-to-cost ratio, 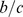. In order to satisfy the weak selection condition 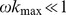, 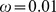 for SW-I, SW-II and RD (Figure 4A) and 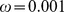 for SF (Figure 4B). The theoretical predictions present a good approximation to the numerical results, i.e. the relationship between the benefit-to-cost ratio and the harmonic mean of the degree distribution in a heterogeneous graph determines whether cooperation will be favored by natural selection.

## Discussion

Ohtsuki et al. [49] argued that the following simple rule that arises from their theoretical analysis of the homogeneous graph should also hold in general: the fixation probability of a single cooperator is larger than 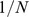 if 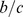 is larger than the average degree of the graph. Later, Tomohiko [54] provided a more accurate approximation based on the average degree of the nearest neighbors. In this paper, we find that the fixation probability of a cooperator depends crucially on its degree. In our theoretical framework, a new state variable 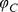 is used to measure the time evolution of cooperation in a heterogeneous graph, which is the proportion of the total degree of cooperators in the total directed edges, instead of the frequency of cooperators in the population.

If all individuals have the same number of neighbors, then 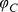 equals the frequency of cooperators and so Ohtsuki et al.'s method is a special case of our model. On the other hand, for the expected payoff of an individual, two cases are considered, called the fixed cost per game and the fixed cost per individual, respectively. In the case of fixed cost per game, a cooperator pays a cost 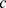 for each of its neighbors to get a benefit 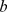; and in the case of fixed cost per individual, a cooperator with 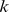 neighbors pays a cost 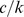 for each of its neighbors to get a benefit 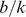, i.e. a cooperator will provide a combined fixed benefit 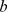 to its all neighbors and pay a fixed cost 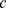 equally shared by its all neighbors. For the evolution of cooperation in a heterogeneous graph, our main results show that (i) under neutral selection, the fixation probability of a single cooperator depends linearly on the number of its neighbors, i.e. the more neighbors a single cooperator has, the larger its fixation probability; (ii) under weak selection, cooperation is favored by natural selection if the benefit-to-cost ratio, 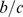, is larger than the average degree, 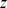, in the case of fixed cost per game or if the benefit-to-cost ratio, 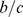, is larger than the harmonic mean of the degree distribution, 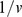, in the case of fixed cost per individual; and (iii) the heterogeneity of a graph is measured by the square of the relative deviation of the degree distribution, 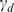, and under weak selection, the effect of selection will decrease with the increase of 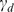.

Our analysis provides an insight for understanding the evolution of cooperation in a heterogeneous graph. In the case of fixed cost per game, Ohtsuki et al.'s simple rule [49] is still valid on average. However, numerical simulations in their paper show that the fixation probabilities of cooperators in heterogeneous graphs such as the scale-free graph are lower than the theoretical prediction. There are two possible reasons. On the one hand, the strength of selection decreases as the graph heterogeneity increases, which implies that the fixation probability is more sensitive to random effects in a strongly heterogeneous graph. Therefore, compared to homogenous graphs, more simulation periods are needed to calculate the fixation probability. In our paper, fixation probabilities of cooperators were found by using either 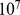 or 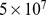 runs (which is ten times the number of runs used in Ohtsuki et al.'s simulations) and our simulation results are closer to the theoretical prediction. On the other hand, the selection intensity (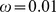) in Ohtsuki et al.'s simulations is not weak enough. In a heterogeneous graph, a cooperator with many neighbors pays significantly higher than a cooperator with few neighbors. This makes the survival of large degree cooperators very difficult.

In a real population, as pointed out by Santos et al. [33], there is no reason for every cooperator to contribute the same amount to each game in which he/she participates. Santos et al. [33] then introduced the concept of fixed cost per individual. When the contribution per individual is fixed, their simulations showed that there is an impressive boost of cooperation for scale-free graphs under the case of fixed cost per individual compared to the case of fixed cost per game. In fact, the promotion of cooperation is due to the " symmetry breaking" of the game [35]. That is, the payoff difference between cooperator and defector in a single game is no longer 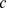, but inversely proportional to the number of games each player plays. This then gives an evolutionary advantage to large degree cooperators. Our theoretical results confirm Santos et al.'s finding. Since the harmonic mean of the degree distribution decreases as the graph heterogeneity increases, social diversity promotes the emergence of cooperation.

Ohtsuki et al. [49] indicated that in social networks, people might have a substantial number of connections, but only very few of them are strong. Hence, they thought that the ‘effective’ average degree of many relevant networks could be small, thereby making selection of cooperation on graphs a powerful option. Obviously, for the case of fixed cost per game, our results provide a reasonable theoretical explanation for Ohtsuki et al.'s conjecture. In fact, the low connectivity on average is more favorable for the emergence of cooperation for the case of fixed cost per individual since the harmonic mean of the degree distribution is less than the average degree in general. It is important to point out that, although cooperation will be favored by natural selection when the average degree is low, the fixation probability of a single given cooperator increases when it has more neighbors.

Graph heterogeneity should be one of the most important characteristics of natural and social networks. Our study analyzes the effect of graph heterogeneity under weak selection only, and a further question is how to explain the effect of strong selection. Evolution of cooperation under strong selection in a heterogeneous graph have been considered by several authors [30]–[34], [55]–[58]. Unfortunately, our theoretical methods cannot be easily extended to the situation with strong selection. In fact, it has been shown that analytical predictions obtained in the pair approximation leads to an apparent contradiction with simulation results even in regular graphs [11], [59]–[61]. In our opinion, other theoretical methods such as the " macro-dynamics" approach developed by Pinheiro et al. [57]–[58] are then better able method to explain the evolution of cooperation under strong selection in a heterogeneous graph.

## Supporting Information

Supporting Information S1 **Supporting Information (containing one table) for Evolution of cooperation in a heterogeneous graph: Fixation probabilities under neutral selection.**
(PDF)

## References

[1] Axelord R (1984) The Evolution of Cooperation. New York:Basic Books.

[2] Nowak MA (2006) Evolutionary Dynamics. Cambridge:Harvard University Press.

[3] Nowak MA, Sigmund K (2007) How population cohere: Five rules for cooperation. In MayR, editor. Theoretical Ecology: Principle and Applications (Third Edition). Oxford:Oxford University Press.

[4] Sherratt TN, Wilkinson DM (2009) Big Questions in Ecology and Evolution. Oxford:Oxford University Press.

[5] Nowak MA (1992) Evolutionary games and spatial chaos. Nature 359: 826–829.

[6] Nowak MA, May RM (1993) The spatial dilemmas of evolution. International Journal of Bifurcation and Chaos 3: 35–78.

[7] Nowak MA, Bonhoeffer S, May RM (1994) Spatial games and the maintenance of cooperation. Proceedings of the National Academy of Sciences of the USA 91: 4877–4881. 8197150 10.1073/pnas.91.11.4877 PMC43892

[8] Szabó G, Tőke C (1998) Evolutionary Prisoner's Dilemma game on a square lattice. Physical Review E 58: 69–73.

[9] Szabó G, Hauert C (2002) Phase transitions and volunteering in spatial public goods games. Physics Review Letters 89: 118101. 10.1103/PhysRevLett.89.118101 12225171

[10] Hauert C, Doebeli M (2004) Spatial structure often inhibits the evolution of cooperation in the snowdrift game. Nature 428: 643–646. 15074318 10.1038/nature02360

[11] Szabó G, Fáth G (2007) Evolutionary games on graphs. Physics Reports 446: 97–216.

[12] Wang Z, Wang L, Yin ZY, Xia CY (2012) Inferring reputation promotes the evolution of cooperation in spatial social dilemma games. PLoS ONE 7: e40218. 22808120 10.1371/journal.pone.0040218 PMC3392274

[13] Shigaki K, Tanimoto J, Wang Z, Kokubo S, Hagishima A, et al (2012) Referring to the social performance promotes cooperation in spatial prisoner's dilemma games. Physics Review E 86: 031141. 10.1103/PhysRevE.86.031141 23030900

[14] Wang Z, Szolnoki A, Perc M (2012) If players are sparse social dilemmas are too: Importance of percolation for evolution of cooperation. Scientific Reports 2: 369. 22511999 10.1038/srep00369 PMC3328045

[15] Traulsen A, Semmann D, Sommerfeld RD, Krambeck HJ, Milinski M (2010) Human strategy updating in evolutionary games. Proceedings of the National Academy of Sciences of the USA 91: 4877–4881. 10.1073/pnas.0912515107 PMC2840356 20142470

[16] Grujić J, Fosco C, Araujo L, Cuesta JA, Sánchez A (2010) Social experiments in the mesoscale: Humans playing a spatial Prisoner's Dilemma. PLoS ONE 5: e13749. 21103058 10.1371/journal.pone.0013749 PMC2980480

[17] Grujić J, Röhl T, Semmann D, Milinski M, Traulsen A (2012) Consistent strategy updating in spatial and non-spatial behavioral experiments does not promote cooperation in social networks. PLoS ONE 7: e47718. 23185242 10.1371/journal.pone.0047718 PMC3501511

[18] Albert R, Jeong H, Barabási AL (1999) Internet: Diameter of the world-wide web. Nature 401: 130–131.

[19] Amaral LAN, Scala A, Barthélémy M, Stanley HE (2000) Classes of small-world networks. Proceedings of the National Academy of Sciences of the USA 97: 11149–11152. 11005838 10.1073/pnas.200327197 PMC17168

[20] Jeong H, Tombor B, Albert R, Ottval ZN, Barabási AL (2000) The large-scale organization of metabolic networks. Nature 407: 651–654. 11034217 10.1038/35036627

[21] Liljerso F, Edling CR, Amaral LAN, Stanley HE, Åberg Y (2001) The web of human sexual contacts. Nature 411: 907–908. 11418846 10.1038/35082140

[22] Dorogotsev SN, Mendes JFF (2003) Evolution of Networks: From Biological Nets to the Internet and WWW. Oxford:Oxford University Press.

[23] Jackson MO (2008) Social and Economic Networks.Princeton:Princeton University Press.

[24] Erdös P, Rényi A (1960) On the evolution of random graphs. Publications of the Mathematical Institute of the Hungarian Academy of Sciences 5: 17–61.

[25] Newman MEJ, Strogatz SH, Watts DJ (2001) Random graphs with arbitrary degree distribution and their applications. Physical Review E 64: 026118. 10.1103/PhysRevE.64.026118 11497662

[26] Watts DJ, Strogatz SH (1998) Collective dynamics of small-world networks. Nature 393: 440–442. 9623998 10.1038/30918

[27] Watts DJ (1999) Small Worlds. Princeton:Princeton University Press.

[28] Barabási AL, Albert R (1999) Emergence of scaling in random networks. Science 15: 509–512. 10.1126/science.286.5439.509 10521342

[29] Albert R, Barabási AL (2002) Statistical mechanics of complex networks. Reviews of Modern Physics 74: 47–98.

[30] Santos FC, Pacheco JM, Lenaerts T (2006) Evolutionary dynamics of social dilemmas in the structured heterogeneous populations. Proceedings of the National Academy of Sciences of the USA 103: 3490–3494. 16484371 10.1073/pnas.0508201103 PMC1413882

[31] Santos FC, Rodrigues JF, Pacheco JM (2006) Graph topology plays a determinant role in the evolution of cooperation. Proceedings of the Royal Society B: Biological Sciences 273: 51–55. 16519234 10.1098/rspb.2005.3272 PMC1560002

[32] Fu F, Liu LH, Wang L (2007) Evolutionary Prisoner's Dilemma on heterogeneous Newman-Watts small-world network. The European Physical Journal B 56: 367–372.

[33] Santos FC, Santos MD, Pacheco JM (2008) Social diversity promotes the emergence of cooperation in the public goods game. Nature 454: 213–216. 18615084 10.1038/nature06940

[34] Devlin S, Treloar T (2009) Evolution of cooperation through the heterogeneity of random networks. Physical Review E 79: 016107. 10.1103/PhysRevE.79.016107 19257107

[35] Pacheco JM, Pinheiro FL, Santos FC (2009) Population structure induces a symmetry breaking favoring the emergence of cooperation. PLoS Computational Biology 5: e1000596. 20011116 10.1371/journal.pcbi.1000596 PMC2782104

[36] Santos FC, Pinheiro FL, Lenaerts T, Pacheco JM (2012) The role of diversity in the evolution of cooperation. Journal of Theoretical Biology 299: 88–96. 21930134 10.1016/j.jtbi.2011.09.003

[37] Szolnoki A, Perc M, Danku Z (2008) Towards effective payoffs in the prisoner's dilemma game on scale-free networks. Physica A 387: 2075–2082.

[38] Assenza S, Gómez-Gardeñes J, Latora V (2008) Enhancement of cooperation in highly clustered scale-free networks. Physical Review E 78: 017101. 10.1103/PhysRevE.78.017101 18764081

[39] Poncela J, Gómez-Gardeñes J, Floría LM, Moreno Y, Sánchez A (2009) Cooperative scale-free networks despite the presence of defector hubs. Europhysics Letters 88: 38003.

[40] Poncela J, Gómez-Gardeñes J, Moreno Y, Floría LM (2010) Cooperation in the Prisoner's Dilemma game in random scale-free graphs. International Journal of Bifurcation and Chaos 20: 849–857.

[41] Tanimoto J, Yamauchi A (2010) Does “game participation cost” affect the advantage of heterogeneous networks for evolving cooperation? Physica A 389: 2284–2289.

[42] Perc M, Gómez-Gardeñes J, Szolnoki A, Floría LM, Moreno Y (2013) Evolutionary dynamics of group interactions on structured populations: a review. Journal of The Royal Society Interface 10: 20120997. 10.1098/rsif.2012.0997 PMC3565747 23303223

[43] Zimmermann MG, Eguluz V, San Miguel M (2004) Coevolution of dynamical states and interactions in dynamic networks. Physical Review E 69: 065102 (R).. 10.1103/PhysRevE.69.065102 15244650

[44] Pacheco JM, Traulsen A, Nowak MA (2006) Coevolution of strategy and structure in complex networks with dynamical linking. Physical Review Letters 97: 258103. 17280398 10.1103/PhysRevLett.97.258103 PMC2430061

[45] Szolnoki A, Perc M, Danku Z (2008) Making new connections towards cooperation in the Prisoner's Dilemma game. Europhysics Letters 84: 50007.

[46] Perc M, Szolnoki A (2010) Coevolutionary games-A mini review. Biosystems 99: 109–125. 19837129 10.1016/j.biosystems.2009.10.003

[47] Zhang CY, Zhang JL, Xie GM, Wang L, Perc M (2011) Evolution of interactions and cooperation in the spatial Prisoner's Dilemma game. PLoS ONE 6: e26724. 22066006 10.1371/journal.pone.0026724 PMC3204981

[48] Segbroeck SV, Santos FC, Lenaerts T, Pacheco JM (2011) Selection pressure transforms the nature of social dilemmas in adaptive networks. New Journal of Physics 13: 013007.

[49] Ohtsuki H, Hauert C, Lieberman E, Nowak MA (2006) A simple rule for the evolution of cooperation on graphs and social networks. Nature 441: 502–505. 16724065 10.1038/nature04605 PMC2430087

[50] Newman M, Barabási AL,Watts DJ (2006) The Structure and Dynamics of Networks.Princeton:Princeton University Press.

[51] Ohtsuki H, Nowak MA (2006) The replicator equation on graphs. Journal of Theoretical Biology 243: 68–97. 10.1016/j.jtbi.2006.06.004 PMC2430083 16860343

[52] Ohtsuki H, Nowak MA (2009) Evolutionary stability on graphs. Journal of Theoretical Biology 251: 698–707. 10.1016/j.jtbi.2008.01.005 PMC2430060 18295801

[53] Ewens WJ (2004) Mathematical Population Genetics: I. Theoretical Introduction. Berlin:Springer.

[54] Tomohiko K (2011) A condition for cooperation in a game on complex networks. Journal of Theoretical Biology 269: 224–233. 21044635 10.1016/j.jtbi.2010.10.033

[55] Gómez-Gardeñes J, Campillo M, Floría LM, Moreno Y (2007) Dynamical organization of cooperation in complex topologies. Physical Review Letters 98: 108103. 17358570 10.1103/PhysRevLett.98.108103

[56] Wang Z, Szolnoki A, Perc M (2012) Evolution of public cooperation on interdependent networks: The impact of biased utility functions. Europhysics Letters 97: 48001.

[57] Pinheiro FL, Santos FC, Pacheco JM (2012) How selection pressure changes the nature of social dilemmas in structured populations. New Journal of Physics 14: 073035.

[58] Pinheiro FL, Santos FC, Pacheco JM (2012) From local to global dilemmas in social networks. PLoS ONE 7: e32114. 22363804 10.1371/journal.pone.0032114 PMC3283728

[59] Szabó G, Vukov J, Szolnoki A (2005) Phase diagrams for an evolutionary prisoner's dilemma game on two-dimensional lattices. Physical Review E 72: 047107. 10.1103/PhysRevE.72.047107 16383580

[60] Vukov J, Szabó G, Szolnoki A (2006) Cooperation in the noisy case: Prisoner's dilemma game on two types of regular random graphs. Physical Review E 73: 067103. 10.1103/PhysRevE.73.067103 16907030

[61] Szabó G, Vukov J, Szolnoki A (2009) Selection of noise level in strategy adoption for spatial social dilemmas. Physical Review E 80: 056112. 10.1103/PhysRevE.80.056112 20365048

[62] Nobari S, Lu XS, Karras P, Bressan S (2011) Fast random graph generation. Proceedings of the 14th International Conference on Extending Database Technology 331–342.

