# Supplementary material for: Evolution of Cooperation in a Heterogeneous Graph: Fixation Probabilities under Weak Selection
Source: PLoS One. 2013 Jun 20;8(6):e66560. doi: 10.1371/journal.pone.0066560 (PMC3688584; doi:10.1371/journal.pone.0066560)
Supplement: Supporting Information S1 — Supporting Information (containing one table) for Evolution of cooperation in a heterogeneous graph: Fixation probabilities under neutral selection. (PDF) [file pone.0066560.s001.pdf]

Supporting Information for "Evolution of cooperation in a heterogeneous graph: Fixation probabilities under neutral selection "

Cong Li[1], Boyu Zhang[2] *and Ross Cressman[3] †, Yi Tao[1]

[1]Key Lab of Animal Ecology and Conservational Biology

Institute of Zoology, Chinese Academy of Sciences

Beijing, P.R. China

[2]School of Mathematical Sciences, Beijing Normal University

Beijing, P.R. China

[3]Department of Mathematics, Wilfrid Laurier University

Waterloo, ON Canada

*Corresponding Author: zhangboyu5507@gmail.com

†Corresponding Author: rcressman@wlu.ca

Following the social network model developed by Ohtsuki et al. [1], we consider a connected graph with N vertices and degree distribution $p(k)$. That is, $p(k)$ denotes the frequency of vertices (or individuals) with k neighbors for $k = 1, 2, \cdots$, or, alternatively, $p(k)$ is the probability that a vertex has k neighbors [2]. The individual at each vertex is either a cooperator in interactions with all of the neighbors or a defector. To describe the time evolution of cooperation through these interactions, we provide some basic assumptions and terminology from graph theory [3] in the following definition.

Definitions

Suppose that G is a directed graph. For this graph, we define:

(i) $\mathbf{I} = \{1, 2, \cdots, N\}$ denotes the set of vertices (individuals). In particular, the total population size is fixed;

(ii) Let (i, j) denote a directed edge between two different vertices i and j for $i, j \in \mathbf{I}$ and let $\mathbf{E}$ be the subset of $\{(i, j) : i, j \in \mathbf{I}\}$ that denotes the set of all possible directed edges in the graph. For our main goal, we assume that, if (i, j) is a directed edge of G, then (j, i) must also be a directed edge of G;

(iii) The state (or strategy) of vertex i for $i \in \mathbf{I}$ is denoted by s_i with $s_i \in \{C, D\}$, where C and D denote cooperation and defection, respectively.

(iv) The degree of vertex i is denoted by k_i, i.e. the number of the i-th individual's neighbors, which can also be defined as $k_i = \sum\limits_{j \in \mathbf{I}} \vartheta_{ij}$ where $\vartheta_{ij} = 1$ if $(i, j) \in \mathbf{E}$ and $\vartheta_{ij} = 0$ if $(i, j) \notin \mathbf{E}$, and let $k_{i,C}$ denote the number of its neighbors with strategy C and $k_{i,D}$ the number of its neighbors with strategy D, i.e. $k_i = k_{i,C} + k_{i,D}$;

(v) The average degree of the graph is $z = \sum\limits_{i \in \mathbf{I}} k_i / N$, or $z = \sum_k kp(k)$. It is the expected number of neighbors of an individual chosen at random;

(vi) For $X, Y \in \{C, D\}$, define $\Phi_{XY} = \sum\limits_{(i,j) \in \mathbf{E}} \psi_{ij}$ where $\psi_{ij} = 1$ if $s_i = X$ and $s_j = Y$, and $\psi_{ij} = 0$ if otherwise. That is, for a given strategy pair (X, Y), Φ_{XY} denotes the number of directed edges (i, j) with $s_i = X$ and $s_j = Y$ for $(i, j) \in \mathbf{E}$, and we have $\Phi_{XY} = \Phi_{YX}$;

(vii) Let $\phi_{XY} = \frac{\Phi_{XY}}{\Phi_{CC}+\Phi_{CD}+\Phi_{DC}+\Phi_{DD}} = \Phi_{XY}/zN$, which denotes the proportion of directed edges with strategy pair (X, Y) in the total set of directed edges;

(viii) The marginal frequency of the state X is denoted by $\phi_X = \phi_{XC} + \phi_{XD}$ with $X \in \{C, D\}$, i.e. ϕ_C (ϕ_D) denotes the proportion of the directed edges starting from cooperators (defectors) in the set of directed edges. Clearly, ϕ_C and ϕ_D can be also expressed as $\phi_C = \sum_{s_i=C} k_i/zN$ and $\phi_D = \sum_{s_i=D} k_i/zN$, respectively, for $i \in \mathbf{I}$. Notice that, if all vertices have the same degree, then ϕ_C and ϕ_D exactly equal the frequencies of cooperators and defectors in the population, respectively;

(ix) For $X, Y \in \{C, D\}$, the conditional probability that a neighbor of a vertex (or an individual) with strategy Y is a X-individual is given by $q_{X|Y} = \phi_{YX}/\phi_Y$.

There are many relationships among the notations summarized in the above definition. For instance, $q_{X|Y}$ and ϕ_{XY} can be also expressed as $q_{X|Y} = \sum_{s_i=Y} k_{i,X} / \sum_{s_i=Y} k_i$ and $\phi_{XY} = \sum_{s_i=X} k_{i,Y} / \sum_i k_i$ (or $\phi_{XY} = \sum_{s_i=X} k_{i,Y}/zN$), respectively, for $X, Y \in \{C, D\}$. We also have that $\phi_C + \phi_D = 1$, $q_{C|X} + q_{D|X} = 1$, $\phi_{XY} = \phi_X \cdot q_{Y|X}$ and $\phi_{CD} = \phi_{DC}$. On the other hand, if we use θ_C and θ_D to denote the frequencies of strategies C and D in the total population, respectively, then $\theta_C + \theta_D = 1$. Furthermore, with $\phi_{k,C}$ ($\phi_{k,D}$) the proportion of vertices with degree k that are in strategy C (D), ϕ_C, ϕ_D, θ_C and θ_D can be expressed as

$$
\begin{aligned}
\phi_C &= \frac{\sum_k k\ \phi_{k,C}\ N\ p(k)}{zN} = \frac{1}{z}\sum_k k\ p(k)\ \phi_{k,C}\ , \\
\phi_D &= \frac{\sum_k k\ \phi_{k,D}\ N\ p(k)}{zN} = \frac{1}{z}\sum_k k\ p(k)\ \phi_{k,D}\ , \\
\theta_C &= \sum_k \phi_{k,C}\ p(k)\ , \\
\theta_D &= \sum_k \phi_{k,D}\ p(k)\ .
\end{aligned}
\tag{S1}
$$

For the 'death-birth' updating process [1,4], assume that an individual is randomly chosen to die, and its neighbors compete for the empty site proportional to their fitness. For a randomly chosen defector, let f_C denote the fitness of its neighbors with strategy C

and f_D the fitness of its neighbors with strategy D, which are given by $f_C = (1-\omega)+\omega h_C$ and $f_D = (1-\omega)+\omega h_D$, respectively, where the parameter ω measures the intensity of selection with $0 \leq \omega \leq 1$, and h_C (h_D) is the expected payoff of each neighbor with strategy C (D). Similarly, for a cooperator, let g_C denote the fitness of its neighbors with strategy C and g_D the fitness of its neighbors with strategy D, which are $g_C = (1-\omega)+\omega l_C$ and $g_D = (1-\omega)+\omega l_D$, respectively, where l_C (l_D) is the expected payoff of the neighbors with strategy C (D).

When a cooperator provides a benefit b at a cost of c to each of its neighbor (i.e. the case of fixed cost per game in the main text), these expected payoffs are

$$\begin{aligned} h_C &= \sum_{\tilde{k}} p(\tilde{k})\Big[(\tilde{k}-1)\big(q_{C|C}\ (b-c)+q_{D|C}\ (-c)\big)-c\Big] \\ &= (z-1)\ q_{C|C}\ b - zc\ , \\ h_D &= \sum_{\tilde{k}} p(\tilde{k})(\tilde{k}-1)\ q_{C|D}\ b \\ &= (z-1)\ q_{C|D}\ b\ . \end{aligned} \tag{S2}$$

and

$$\begin{aligned} l_C &= \sum_{\tilde{k}} p(\tilde{k})\Big[(\tilde{k}-1)\big(q_{C|C}\ (b-c)+q_{D|C}\ (-c)\big)+(b-c)\Big] \\ &= (z-1)\ q_{C|C}\ b + b - zc\ , \\ l_D &= \sum_{\tilde{k}} p(\tilde{k})(\tilde{k}-1)\ q_{C|D}\ b + b \\ &= (z-1)\ q_{C|D}\ b + b\ . \end{aligned} \tag{S3}$$

Notice that the expected payoffs in Eq.(S3) differ from the corresponding ones in Eq.(S2) by the benefit b that the focal cooperator provides each of its neighbor.

In the case of fixed cost per individual, a cooperator with degree k provides a benefit b/k at a cost of c/k to each of its neighbor [5]. Thus, the expected benefit from a randomly chosen C-neighbor becomes $\sum_{\tilde{k}}(b/\tilde{k})p(\tilde{k})$ (instead of b). The analogues of Eqs (S2) and

(S3) are then

$$h_C = (z-1)q_{C|C}\, vb - c\,,$$
$$h_D = (z-1)q_{C|D}\, vb\,, \tag{S4}$$

and

$$l_C = (z-1)q_{C|C}\, vb + \frac{b}{k} - c\,,$$
$$l_D = (z-1)q_{C|D}\, vb + \frac{b}{k} \tag{S5}$$

where $v = \sum_{\tilde{k}}(1/\tilde{k})p(\tilde{k})$.

Diffusion approximation

From the binomial theorem, if a defector with degree k is randomly chosen to die, then the probability that this individual has exactly k_C neighbors with strategy C and k_D neighbors with strategy D is $\frac{k!}{k_C!k_D!}\left(q_{C|D}\right)^{k_C}\left(q_{D|D}\right)^{k_D}$. Thus, the probability that the change of ϕ_C equals exactly $\Delta\phi_C = k/zN$ in one time step is

$$\Pr\left(\Delta\phi_C = \frac{k}{zN}\right) = p(k)\phi_{k,D} \sum_{k_C+k_D=k} \frac{k!}{k_C!k_D!}\left(q_{C|D}\right)^{k_C}\left(q_{D|D}\right)^{k_D} \frac{k_C f_C}{k_C f_C + k_D f_D}\,, \tag{S6}$$

and the probability that the change of ϕ_{CC} equals exactly $\Delta\phi_{CC} = 2k_C/zN$ is

$$\Pr\left(\Delta\phi_{CC} = \frac{2k_C}{zN}\right) = \sum_{k\geq k_C}\Bigg[p(k)\phi_{k,D}\frac{k!}{k_C!(k-k_C)!}\left(q_{C|D}\right)^{k_C}\left(q_{D|D}\right)^{k_D} \times \frac{k_C f_C}{k_C f_C + (k-k_C) f_D}\Bigg]. \tag{S7}$$

Similarly, if a cooperator with degree k is randomly chosen to die, then the probability that the change of ϕ_C equals exactly $\Delta\phi_C = -k/zN$ is

$$\Pr\left(\Delta\phi_C = -\frac{k}{zN}\right) = p(k)\phi_{k,C} \sum_{k_C+k_D=k} \frac{k!}{k_C!k_D!}\left(q_{C|C}\right)^{k_C}\left(q_{D|C}\right)^{k_D} \frac{k_D g_D}{k_C g_C + k_D g_D}\,, \tag{S8}$$

and the probability that the change of ϕ_{CC} equals exactly $\Delta\phi_{CC} = -2k_C/zN$ is

$$\Pr\left(\Delta\phi_{CC} = -\frac{2k_C}{zN}\right) = \sum_{k\geq k_C}\left[p(k)\phi_{k,C}\frac{k!}{k_C!(k-k_C)!}\left(q_{C|C}\right)^{k_C}\left(q_{D|C}\right)^{k_D} \times \frac{(k-k_C)g_D}{k_C g_C + (k-k_C)g_D}\right] . \quad \text{(S9)}$$

Notice that, under weak selection (i.e. $k_{\max}\omega \ll 1$), the terms $\frac{k_C f_C}{k_C f_C + k_D f_D}$ and $\frac{k_D g_D}{k_C g_C + k_D g_D}$ can be approximated as

$$\begin{aligned} \frac{k_C f_C}{k_C f_C + k_D f_D} &= \frac{k_C}{k} + \omega\frac{k_C k_D}{k^2}\left(\frac{df_C}{d\omega} - \frac{df_D}{d\omega}\right) + \mathcal{O}(\omega^2) \\ &= \frac{k_C}{k} + \omega\frac{k_C k_D}{k^2}\left(h_C - h_D\right) + \mathcal{O}(\omega^2) , \end{aligned} \quad \text{(S10)}$$

and

$$\begin{aligned} \frac{k_D g_D}{k_C g_C + k_D g_D} &= \frac{k_D}{k} + \omega\frac{k_C k_D}{k^2}\left(\frac{dg_D}{d\omega} - \frac{dg_C}{d\omega}\right) + \mathcal{O}(\omega^2) \\ &= \frac{k_D}{k} + \omega\frac{k_C k_D}{k^2}\left(l_D - l_C\right) + \mathcal{O}(\omega^2) . \end{aligned} \quad \text{(S11)}$$

For convenience, let $\Delta_1 = h_C - h_D$ and $\Delta_2 = l_D - l_C$. Thus, the term $\sum\limits_{k_C+k_D=k}\frac{k!}{k_C!k_D!}\left(q_{C|D}\right)^{k_C} \times \left(q_{D|D}\right)^{k_D}\frac{k_C f_C}{k_C f_C + k_D f_D}$ in (S6) can be expressed as

$$\begin{aligned} &\sum_{k_C+k_D=k}\frac{k!}{k_C!k_D!}\left(q_{C|D}\right)^{k_C}\left(q_{D|D}\right)^{k_D}\frac{k_C f_C}{k_C f_C + k_D f_D} \\ =& \sum_{k_C+k_D=k}\frac{k!}{k_C!k_D!}\left(q_{C|D}\right)^{k_C}\left(q_{D|D}\right)^{k_D}\left[\frac{k_C}{k} + \omega\frac{k_C k_D}{k^2}\Delta_1\right] + \mathcal{O}(\omega^2) \\ =& \left(1+\omega\Delta_1\right)\frac{1}{k}\sum_{k_C+k_D=k} k_C\frac{k!}{k_C!k_D!}\left(q_{C|D}\right)^{k_C}\left(q_{D|D}\right)^{k_D} \\ & -\frac{\omega\Delta_1}{k^2}\sum_{k_C+k_D=k} k_C^2\frac{k!}{k_C!k_D!}\left(q_{C|D}\right)^{k_C}\left(q_{D|D}\right)^{k_D} + \mathcal{O}(\omega^2) \\ =& \left(1+\omega\Delta_1\right)\frac{1}{k}\cdot k\ q_{C|D} - \frac{\omega\Delta_1}{k^2}\cdot k\ q_{C|D}\left(k\ q_{C|D} + q_{D|D}\right) + \mathcal{O}(\omega^2) \\ =& q_{C|D} + \omega\Delta_1\ q_{C|D}\ q_{D|D}\left(1-\frac{1}{k}\right) + \mathcal{O}(\omega^2) , \end{aligned} \quad \text{(S12)}$$

and, similarly, the term $\sum\limits_{k_C+k_D=k}\frac{k!}{k_C!k_D!}\left(q_{C|C}\right)^{k_C}\left(q_{D|C}\right)^{k_D}\frac{k_D g_D}{k_C g_C + k_D g_D}$ in (S8) can be ex-

pressed as

$$
\begin{aligned}
&\sum_{k_C+k_D=k} \frac{k!}{k_C!k_D!}\big(q_{C|C}\big)^{k_C}\big(q_{D|C}\big)^{k_D}\frac{k_D g_D}{k_C g_C + k_D g_D} \\
&= \quad q_{D|C} + \omega\Delta_2\, q_{C|C}\, q_{D|C}\left(1-\frac{1}{k}\right) + \mathcal{O}\big(\omega^2\big)\ . \qquad \text{(S13)}
\end{aligned}
$$

Then, the time evolution of ϕ_C is given by

$$
\begin{aligned}
\frac{d\phi_C}{dt} &= \sum_{k=1}^{N-1}\frac{k}{zN}\,\mathrm{Pr}\left(\Delta\phi_C = \frac{k}{zN}\right) + \sum_{k=1}^{N-1}\left(-\frac{k}{zN}\right)\mathrm{Pr}\left(\Delta\phi_C = -\frac{k}{zN}\right) \\
&= \sum_{k=1}^{N-1}\left[\frac{k}{zN}p(k)\phi_{k,D}\left(q_{C|D} + \omega\Delta_1\, q_{C|D}\, q_{D|D}\ \left(1-\frac{1}{k}\right)\right)\right] \\
&\quad -\sum_{k=1}^{N-1}\left[\frac{k}{zN}\ p(k)\phi_{k,C}\left(q_{D|C} + \omega\Delta_2\, q_{C|C}\, q_{D|C}\ \left(1-\frac{1}{k}\right)\right)\right] \\
&\quad +\mathcal{O}\big(\omega^2\big) \\
&= \sum_{k=1}^{N-1}\left[\frac{\omega(k-1)}{zN}p(k)\Big(\Delta_1\phi_{k,D}\, q_{C|D}\, q_{D|D} - \Delta_2\phi_{k,C}\, q_{D|C}\, q_{C|C}\Big)\right] \\
&\quad +\mathcal{O}\big(\omega^2\big) \\
&= \frac{\omega}{zN}\sum_{k=1}^{N-1}\Big[p(k)(k-1)\big(\Delta_1\phi_{k,D}\, q_{C|D}\, q_{D|D} - \Delta_2\phi_{k,C}\, q_{D|C}\, q_{C|C}\big)\Big] \\
&\quad +\mathcal{O}\big(\omega^2\big) \qquad \text{(S14)}
\end{aligned}
$$

since

$$
\begin{aligned}
\phi_C &= \frac{1}{z}\sum_k kp(k)\phi_{k,C}\ , \\
\phi_D &= \frac{1}{z}\sum_k kp(k)\phi_{k,D}
\end{aligned}
$$

in Eq.(S1). Furthermore, the time evolution of ϕ_{CC} is given by

$$
\begin{aligned}
\frac{d\phi_{CC}}{dt} &= \sum_{k_C=1}^{N-1} \frac{2k_C}{zN} \Pr\left(\Delta\phi_{CC} = \frac{2k_C}{zN}\right) + \sum_{k_C=1}^{N-1} \left(-\frac{2k_C}{zN}\right) \Pr\left(\Delta\phi_{CC} = -\frac{2k_C}{zN}\right) \\
&= \sum_{k_C=1}^{N-1} \left[\frac{2k_C}{zN} \sum_{k \geq k_C} p(k)\phi_{k,D} \frac{k!}{k_C!(k-k_C)!} \left(q_{C|D}\right)^{k_C} \left(q_{D|D}\right)^{k-k_C} \frac{k_C}{k}\right] \\
&\quad + \sum_{k_C=1}^{N-1} \left[-\frac{2k_C}{zN} \sum_{k \geq k_C} p(k)\phi_{k,C} \frac{k!}{k_C!(k-k_C)!} \left(q_{C|C}\right)^{k_C} \left(q_{D|C}\right)^{k-k_C} \frac{k-k_C}{k}\right] \\
&\quad + \mathcal{O}(\omega) \\
&= \frac{2}{zN} \sum_{k_C=1}^{N-1} \sum_{k=k_C}^{N-1} \left[k_C \; p(k) \; \phi_{k,D} \frac{k!}{k_C!(k-k_C)!} \left(q_{C|D}\right)^{k_C} \left(q_{D|D}\right)^{k-k_C} \frac{k_C}{k}\right] \\
&\quad - \frac{2}{zN} \sum_{k_C=1}^{N-1} \sum_{k=k_C}^{N-1} \left[k_C \; p(k) \; \phi_{k,C} \frac{k!}{k_C!(k-k_C)!} \left(q_{C|C}\right)^{k_C} \left(q_{D|C}\right)^{k-k_C} \frac{k-k_C}{k}\right] \\
&\quad + \mathcal{O}(\omega) \\
&= \frac{2}{zN} \sum_{k=1}^{N-1} \sum_{k_C=1}^{k} \left[k_C \; p(k) \; \phi_{k,D} \frac{k!}{k_C!(k-k_C)!} \left(q_{C|D}\right)^{k_C} \left(q_{D|D}\right)^{k-k_C} \frac{k_C}{k}\right] \\
&\quad - \frac{2}{zN} \sum_{k=1}^{N-1} \sum_{k_C=1}^{k} \left[k_C \; p(k) \; \phi_{k,C} \frac{k!}{k_C!(k-k_C)!} \left(q_{C|C}\right)^{k_C} \left(q_{D|C}\right)^{k-k_C} \frac{k-k_C}{k}\right] \\
&\quad + \mathcal{O}(\omega) \\
&= \frac{2}{zN} \sum_{k=1}^{N-1} \frac{p(k)\phi_{k,D}}{k} \sum_{k_C=1}^{k} \left[k_C^2 \frac{k!}{k_C!(k-k_C)!} \left(q_{C|D}\right)^{k_C} \left(q_{D|D}\right)^{k-k_C}\right] \\
&\quad - \frac{2}{zN} \sum_{k=1}^{N-1} \frac{p(k)\phi_{k,C}}{k} \sum_{k_C=1}^{k} \left[k_C(k-k_C) \frac{k!}{k_C!(k-k_C)!} \left(q_{C|C}\right)^{k_C} \left(q_{D|C}\right)^{k-k_C}\right] \\
&\quad + \mathcal{O}(\omega) \\
&= \frac{2}{zN} \sum_{k=1}^{N-1} \frac{p(k)\phi_{k,D}}{k} \; k \; q_{C|D} \big(k \; q_{C|D} + q_{D|D}\big) \\
&\quad - \frac{2}{zN} \sum_{k=1}^{N-1} \frac{p(k)\phi_{k,C}}{k} \; k \; q_{C|C} (k-1) \; q_{D|C} + \mathcal{O}(\omega) \\
&= \frac{2}{zN} \sum_{k=1}^{N-1} p(k) \Big[\phi_{k,D} \; q_{C|D} \big(k q_{C|D} + q_{D|D}\big) - \phi_{k,C} \; q_{C|C} \; q_{D|C} (k-1)\Big] \\
&\quad + \mathcal{O}(\omega) \; . \qquad \text{(S15)}
\end{aligned}
$$

Similarly, for each degree k, the time evolution of $\phi_{k,C}$ is given by

$$
\begin{aligned}
\frac{d\phi_{k,C}}{dt} &= \frac{1}{Np(k)}\,\mathrm{Pr}\left(\Delta\phi_{k,C}=\frac{1}{Np(k)}\right)-\frac{1}{Np(k)}\,\mathrm{Pr}\left(\Delta\phi_{k,C}=-\frac{1}{Np(k)}\right)\\
&= \frac{1}{Np(k)}\left(p(k)\phi_{k,D}q_{C|D}-p(k)\phi_{k,C}q_{D|C}\right)+\mathcal{O}(\omega)\\
&= \frac{1}{Np(k)}\left(p(k)\phi_{k,D}\frac{\phi_{DC}}{\phi_D}-p(k)\phi_{k,C}\frac{\phi_{CD}}{\phi_C}\right)+\mathcal{O}(\omega)\\
&= \frac{\phi_{CD}}{N\phi_C\phi_D}((1-\phi_{k,C})\phi_C-\phi_{k,C}(1-\phi_C))+\mathcal{O}(\omega)\\
&= \frac{\phi_{CD}}{N\phi_C\phi_D}(\phi_C-\phi_{k,C})+\mathcal{O}(\omega) \qquad \text{(S16)}
\end{aligned}
$$

Notice that the time evolutions of ϕ_{CC} and $\phi_{k,C}$ include the term ω^0. Thus, for the situation with weak selection (i.e. $\omega \ll 1$), ϕ_{CC} and $\phi_{k,C}$ should be considered to be fast variables compared with ϕ_C. Thus, if we assume that ϕ_{CC} and $\phi_{k,C}$ have reached equilibrium for fixed ϕ_C and ϕ_D, the system dynamics is mainly determined by the dynamics of ϕ_C. From $d\phi_{CC}/dt=0$, we have

$$
\begin{aligned}
&\sum_{k=1}^{N-1} p(k)\Big[\phi_{k,D}\ q_{C|D}\big(kq_{C|D}+q_{D|D}\big)-\phi_{k,C}\ q_{C|C}\ q_{D|C}(k-1)\Big] \ = \ 0\ ,\\
&\sum_{k=1}^{N-1} p(k)\Big[k\Big(\phi_{k,D}\big(q_{C|D}\big)^2-\phi_{k,C}\ q_{C|C}\ q_{D|C}\Big)\\
&\qquad\qquad +\Big(\phi_{k,D}\ q_{C|D}\ q_{D|D}+\phi_{k,C}\ q_{C|C}\ q_{D|C}\Big)\Big] \ = \ 0\ ,\\
&\sum_{k=1}^{N-1} p(k)k\big(\phi_{k,D}\ q_{C|D}\ q_{C|D}-\phi_{k,C}\ q_{D|C}\ q_{C|C}\big)\\
&\qquad = \ -\sum_{k=1}^{N-1} p(k)\big(\phi_{k,D}\ q_{C|D}\ q_{D|D}+\phi_{k,C}\ q_{D|C}\ q_{C|C}\big)\ . \qquad \text{(S17)}
\end{aligned}
$$

and from $d\phi_{k,C}/dt=0$, we get

$$\phi_C-\phi_{k,C}=0.$$

This implies that, under weak selection, $\phi_{k,C}$ and $\phi_{k,D}$ can be approximated by ϕ_C and ϕ_D, respectively, for all possible k. Eq.(S17) then can be simplified as

$$(z-1)\big(q_{C|C}-q_{C|D}\big)=1\ .$$

Thus,

$$\phi_{CD} = \frac{z-2}{z-1}\ \phi_C\big(1-\phi_C\big)\ .$$

Notice also that for both fixed cost per game and fixed cost per individual cases, $\Delta_1 = -\Delta_2$. With these simplifications, $d\phi_C/dt$ becomes

$$\begin{aligned}
\frac{d\phi_C}{dt} &= \frac{\omega\Delta_1}{zN}\sum_{k=1}^{N-1}\Big[p(k)(k-1)\big(\phi_{k,D}\ q_{C|D}\ q_{D|D} + \phi_{k,C}\ q_{C|C}\ q_{D|C}\big)\Big] + \mathcal{O}\big(\omega^2\big) \\
&= \frac{\omega\Delta_1}{zN}\sum_{k=1}^{N-1}\Big[p(k)k\big(\phi_{k,D}\ q_{C|D}\ q_{D|D} + \phi_{k,C}\ q_{C|C}\ q_{D|C}\big) \\
&\qquad -p(k)\big(\phi_{k,D}\ q_{C|D}\ q_{D|D} + \phi_{k,C}\ q_{C|C}\ q_{D|C}\big)\Big] + \mathcal{O}\big(\omega^2\big) \\
&= \frac{\omega\Delta_1}{zN}\sum_{k=1}^{N-1}\Big[p(k)k\big(\phi_{k,D}\ q_{C|D}\ q_{D|D} + \phi_{k,C}\ q_{C|C}\ q_{D|C}\big) \\
&\qquad +p(k)k\big(\phi_{k,D}\ q_{C|D}\ q_{C|D} - \phi_{k,C}\ q_{C|C}\ q_{D|C}\big)\Big] + \mathcal{O}\big(\omega^2\big) \\
&= \frac{\omega\Delta_1}{zN}\sum_{k=1}^{N-1}\Big[p(k)k\phi_{k,D}\ q_{C|D}\ \big(q_{C|D} + q_{D|D}\big)\Big] + \mathcal{O}\big(\omega^2\big) \\
&= \frac{\omega\Delta_1}{N}\phi_D\ q_{C|D} + \mathcal{O}\big(\omega^2\big) \\
&= \frac{\omega\Delta_1}{N}\phi_{CD} + \mathcal{O}\big(\omega^2\big)\ . \qquad \text{(S18)}
\end{aligned}$$

This implies that the expectation of $\Delta\phi_C$ in the time interval $(t, t+\Delta t)$ is given by

$$\mathrm{E}\{\Delta\phi_C\}\ =\ \frac{\omega\Delta_1}{N}\phi_{CD}\Delta t + \mathcal{O}\big(\omega^2\big)\ \simeq \tau(\phi_C)\Delta t$$

where $\tau(\phi_C) = \frac{\omega\Delta_1}{N}\phi_{CD}$.

From $d\phi_{CC}/dt = 0$ and (S17), we have that

$$\begin{aligned}
&\sum_{k=1}^{N-1} p(k)k\big(\phi_{k,D}\; q_{C|D}\; q_{C|D} - \phi_{k,C}\; q_{C|C}\; q_{D|C}\big) \\
&\qquad = -\sum_{k=1}^{N-1} p(k)\big(\phi_{k,D}\; q_{C|D}\; q_{D|D} + \phi_{k,C}\; q_{C|C}\; q_{D|C}\big)\ , \\
&z\phi_{CD}\big(q_{C|C} - q_{C|D}\big) = \sum_{k=1}^{N-1} p(k)\big(\phi_{k,D}\; q_{C|D}\; q_{D|D} + \phi_{k,C}\; q_{C|C}\; q_{D|C}\big) \\
\Rightarrow\quad &z\big(q_{C|C} + q_{D|D} - 1\big) = \frac{q_{C|D}\; q_{D|D}}{\phi_{CD}} \sum_{k=1}^{N-1} \phi_{k,D}p(k) + \frac{q_{C|C}\; q_{D|C}}{\phi_{CD}} \sum_{k=1}^{N-1} \phi_{k,C}p(k) \\
&\qquad = q_{D|D}\frac{\sum_{k=1}^{N-1} \phi_{k,D}p(k)}{\phi_D} + q_{C|C}\frac{\sum_{k=1}^{N-1} \phi_{k,C}p(k)}{\phi_C}\ . \qquad \text{(S19)}
\end{aligned}$$

Thus, the variance of $\Delta\phi_C$ in the time interval $(t, t + \Delta t)$ is

$$\begin{aligned}
\mathrm{Var}\{\Delta\phi_C\} &= \mathrm{E}\big\{\Delta\phi_C^2\big\} - \mathrm{E}\{\Delta\phi_C\}^2 \\
&= \sum_{k=1}^{N-1}\left[\left(\frac{k}{zN}\right)^2 \Pr\left(\Delta\phi_C = \frac{k}{zN}\right)\right]\Delta t \\
&\quad + \sum_{k=1}^{N-1}\left[\left(-\frac{k}{zN}\right)^2 \Pr\left(\Delta\phi_C = -\frac{k}{zN}\right)\right]\Delta t - \mathcal{O}\big(\omega^2\big) \\
&= \frac{1}{z^2N^2}\sum_{k=1}^{N-1} k^2 p(k)\big(\phi_{k,C}\; q_{D|C} + \phi_{k,D}\; q_{C|D}\big)\Delta t + \mathcal{O}(\omega) - \mathcal{O}\big(\omega^2\big) \\
&\simeq \mu(\phi_C)\Delta t \qquad \text{(S20)}
\end{aligned}$$

where $\mu(\phi_C) = \frac{1}{z^2N^2}\sum_{k=1}^{N-1} k^2 p(k)\big(\phi_{k,C}\; q_{D|C} + \phi_{k,D}\; q_{C|D}\big)$.

From the theory of diffusion approximation [6], if $\rho(s)$ denote the fixation probability of strategy C with initial $s = \phi_C$ $(t = 0)$, then $\rho(s)$ satisfies

$$\frac{\mu(s)}{2}\frac{d^2\rho(s)}{ds^2} + \tau(s)\frac{d\rho(s)}{ds} = 0\ . \qquad \text{(S21)}$$

For the fixed cost per game case, from Eqs. (S2) and (S19), $\Delta_1 = h_C - h_D = b - zc$ and so $\tau(s) = \frac{\omega}{N}\cdot\frac{z-2}{z-1}\cdot s\big(1-s\big)(b-zc)$. For the fixed cost per individual case, $\Delta_1 = h_C - h_D = vb - c$ and $\tau(s) = \frac{\omega}{N}\cdot\frac{z-2}{z-1}\cdot s\big(1-s\big)(vb-c)$. Similarly, for both cases, $\mu(s) = \frac{2}{N^2}\cdot\frac{\mathrm{E}\{k^2\}}{z^2}\cdot\frac{z-2}{z-1}s\big(1-s\big)$.

Clearly, the solution of Eq.(S21) can be easily expressed as

$$\rho(s) = \frac{e^{\alpha s} - 1}{e^{\alpha} - 1} \ ,$$

where $\alpha = -\omega N\big(z^2/\mathrm{E}\{k^2\}\big)(b - zc)$ for the case of fixed cost per game and $\alpha = -\omega N\big(z^2/\mathrm{E}\{k^2\}\big)(vb - c)$ for the case of fixed cost per individual. Since ω is small, we have the approximation

$$\begin{aligned} \rho(s) &= s + \frac{\omega N}{2} \cdot \frac{z^2}{\mathrm{E}\{k^2\}}\,(b - zc)s(1-s) \\ &= s + \frac{1}{1+\gamma_d} \cdot \frac{\omega N}{2}(b - zc)s(1-s) \end{aligned} \qquad \text{(S22)}$$

for the case of fixed cost per game, and

$$\begin{aligned} \rho(s) &= s + \frac{\omega N}{2} \cdot \frac{z^2}{\mathrm{E}\{k^2\}}\,(vb - c)s(1-s) \\ &= s + \frac{1}{1+\gamma_d} \cdot \frac{\omega N}{2}(vb - c)s(1-s) \end{aligned} \qquad \text{(S23)}$$

for the case of fixed cost per individual, where $\gamma_d = \mathrm{Var}\{k\}/z^2$ and $\mathrm{Var}\{k\} = \mathrm{E}\{k^2\} - z^2$.

Numerical simulations

Generation of Figure 3A

In Figure 3A, we calculate the fixation probability for each b/c in 7 types of random graphs with degree distributions that have broader range of graph heterogeneity [2]. In these graphs, the total population size is $N = 1000$ and the average degree is $z = 20$. So there are $5 \times 7 = 35$ graphs in total which correspond to 35 data points in Figure 3A. These graphs are generated by a variant Erdos-Renyi model [7] and the structures of these graphs change from low heterogeneity to high heterogeneity. At each step, two vertices i and j are randomly chosen with probabilities u_i and u_j (we define them later), respectively. If these two vertices did not connect with each other before, then we can use an edge to connect them. This process is repeated until the total number of edges achieves $zN/2$. However, we here need to guarantee the connectivity of the graph. If not all vertices of the graph are completely connected, we generate the graph again.

In each graph, a vertex i is randomly chosen with probability $u_i = a_i/S$ where a_i is defined as the weight of vertex i and $S = \sum_{i=1}^{N} a_i$. In the simulations, we take

$$a_i = \begin{cases} d & i \leq m \\ 0.3 & m < i \leq N \end{cases},$$

where d and m for 7 types of random graphs are shown in Table S1. In type 1, all vertices are equally weighted which results in the standard random graph. More generally, the degree distribution of this class of random graphs is given by

$$p(k) = \frac{m}{N}\binom{N}{k}(\frac{dz}{S})^k(1-\frac{dz}{S})^{N-k} + \frac{N-m}{N}\binom{N}{k}(\frac{0.3z}{S})^k(1-\frac{0.3z}{S})^{N-k}.$$

Notice that the heterogeneity of the graph is determined by d and m, we can use different values of d and m to generate a series of graphs with different heterogeneities. The simulation results for this family of graphs is shown in Figure 3A.

Table S1

Type	1	2	3	4	5	6	7
d	N/A	1	2	5	10	20	20
m	0	500	100	50	50	50	20

Generation of Figure 3B

In Figure 3B, we calculate the fixation probability for each b/c in 6 types of scale-free graphs with different graph heterogeneities. In these graphs, the total population size is $N = 1000$ and the average degree is $z = 10$. So there are $5 \times 6 = 30$ graphs in total which correspond to 30 data points in Figure 3B.

These scale-free graphs are generated by a variant of Barabási-Albert model. In the standard Barabási-Albert model [8], the probability u that a new vertex will be connected to vertex i depends on the current degree k_i of that vertex, i.e., $u(k_i) = k_i/\sum_{j=1}^{N} k_j$. In this simulation, we consider

$$u(k_i) = \frac{k_i + t}{\sum_{j=1}^{N} k_j + Nt},$$

where $t = 0$ yields the standard (preferential attachment) scale-free graph [8] and $t \to \infty$ leads to random attachment scale-free graph [9]. In Figure 3B, $t = \infty, 30, 15, 5, 2$ and 0, respectively, and the structures of 6 types of scale-free graphs change from low heterogeneity to high heterogeneity.

References

[1] Ohtsuki H, Hauert C, Lieberman E, Nowak MA (2006) A simple rule for the evolution of cooperation on graphs and social networks. Nature 441: 502-505.

[2] Newman MEJ, Strogatz SH, Watts DJ (2001) Random graphs with arbitrary degree distribution and their applications. Physical Review E 64: 026118.

[3] West DB (2001) Introduction to Graph Theory (2nd edition): Prentice Hall.

[4] Ohtsuki H, Nowak MA (2009) Evolutionary stability on graphs. Journal of Theoretical Biology 251: 698-707.

[5] Santos FC, Santos MD, Pacheco JM (2008) Social diversity promotes the emergence of cooperation in the public goods game. Nature 454: 213-216.

[6] Ewens WJ (2004) Mathematical Population Genetics: I. Theoretical Introduction: Springer.

[7] Nobari S, Lu XS, Karras P, Bressan S (2011) Fast random graph generation. Proceedings of the 14th International Conference on Extending Database Technology 331-342.

[8] Barabási AL, Albert R (1999) Emergence of scaling in random networks. Science 15: 509-512.

[9] Barabási AL, Albert R, Jeong H (1999) Mean-field theory for scale-free random networks. Physica A 272: 173-187.
